# Diffuse peritoneal dissemination of intracranial pure germinoma via ventriculoperitoneal shunt

**DOI:** 10.1007/s00234-024-03409-9

**Published:** 2024-06-19

**Authors:** Ryo Kurokawa, Shiori Amemiya, Mariko Kurokawa, Soma Onoda, Hirokazu Takami, Shunsaku Takayanagi, Masako Ikemura, Gakushi Yoshikawa, Osamu Abe

**Affiliations:** 1 Department of Radiology, Graduate School of Medicine, The University of Tokyo, 7-3-1, Hongo, Bunkyo-ku, Tokyo, 113-8655 Japan; 2 Department of Neurosurgery, Graduate School of Medicine, The University of Tokyo, 7-3-1, Hongo, Bunkyo-ku, Tokyo, 113-8655 Japan; 3 Department of Pathology, Graduate School of Medicine, The University of Tokyo, 7-3-1, Hongo, Bunkyo-ku, Tokyo, 113-8655 Japan; 4 Department of Neurosurgery, Showa General Hospital, 8-1-1 Hanakoganei, Kodaira-shi, Tokyo, 187-8510 Japan

**Keywords:** Germinoma, Ventriculoperitoneal shunt, Peritoneal dissemination, Computed tomography, Magnetic resonance imaging

## Abstract

Germinomas frequently cause hydrocephalus, and ventriculoperitoneal shunts (VPS) have been commonly used for their management. Although VPS can potentially serve as a route for peritoneal dissemination of germinomas, the abdominal imaging characteristics of this rare yet important complication remain unknown. In this article, we report the computed tomography imaging findings of diffuse peritoneal dissemination of intracranial germinoma.

## Introduction

Germinomas are malignant germ cell tumors that commonly arise in the pineal and suprasellar regions of the brain, with a male-to-female ratio of 3–4:1 and peak incidence at 11–15 years [1]. Germinomas are known for their high sensitivity to radiation and chemotherapy, leading to favorable outcomes, with a 10-year survival rate of approximately 90% [2]. Meanwhile, germinomas also have a propensity for cerebrospinal fluid dissemination, with reports indicating a 4.5–10% incidence at the time of diagnosis and a high incidence of hydrocephalus in up to 90% of pineal cases [3–5]. Ventriculoperitoneal shunts (VPS) have been commonly used to alleviate hydrocephalus. However, the VPS can potentially serve as a route for peritoneal dissemination of germinomas, and now, endoscopic third ventriculostomy is the recommended therapeutic method with a lower risk of cerebrospinal fluid dissemination [6, 7]. Despite the known risk of peritoneal spread via VPS, the appearance of this complication on abdominal imaging is unknown. Neuroradiologists play a pivotal role in the diagnosis and management of patients with intracranial germinomas and VPS; thus, awareness of the abdominal imaging findings associated with this complication is essential. In this article, we report the imaging findings of diffuse peritoneal dissemination of intracranial pure germinoma via a VPS.

## Methods

We report a case of a patient who developed diffuse peritoneal dissemination via a VPS approximately 20 years after VPS placement for hydrocephalus caused by pineal pure germinoma.

### Clinical summary

**Fig. 1:**
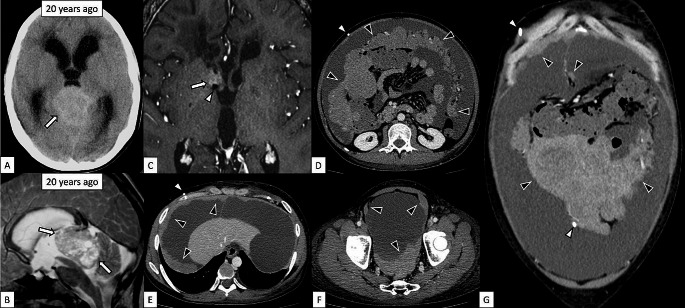
Intracranial pure germinoma and diffuse peritoneal dissemination. **A**, **B**: Nonenhanced CT and T2-weighted sagittal image of the primary pineal pure germinoma 20 years ago. There is a high-attenuated and hyperintense mass with multiple cystic components in the pineal region (**A**, **B**, arrows). **C**: Fat-suppressed 3-dimentional contrast-enhanced T1-weighted imaging of the brain shows a recurrent tumor with contrast enhancement in the right frontal horn (arrow) adjacent to the tip of the VPS (white arrowhead). **D**–**G**: Contrast-enhanced abdominal CT images (**D**–**F**: axial imaging, **G**: coronal imaging) show the VPS (white arrowheads) and diffuse confluent multinodular masses with homogeneous enhancement with approximately 70 Hounsfield Unit, except for the suspected central necrosis in the largest omental mass, along the abdominal wall and greater omentum (black arrowheads). Massive ascites with approximately 20 Hounsfield Unit are also demonstrated

A 32-year-old male patient with a history of pineal pure germinoma, diagnosed and treated 20 years previously, presented with abdominal distension. The initial treatment of the pineal pure germinoma included tumor resection, VPS placement for hydrocephalus, whole-brain radiation (30 Gy), local radiation (20 Gy), and eight cycles of ifosfamide, cisplatin, and etoposide chemotherapy with additional spinal cord radiation (22 Gy) for cauda equina dissemination. Seven years after diagnosis, the patient experienced symptomatic epilepsy due to bilateral frontal lobe recurrences, which were treated with local radiation (30 Gy). The patient continued to take levetiracetam (3,000 mg) orally to control the epilepsy after the episode. Twenty years after diagnosis, an enhancing nodule appeared in the right frontal horn adjacent to the tip of the VPS on magnetic resonance imaging (MRI) and grew over three months (Fig. 1). The patient was referred to our neurosurgery department. Gamma-knife radiosurgery was performed for recurrent lesions. One month later, the patient developed progressive abdominal distension. Contrast-enhanced computed tomotraphy (CT) revealed diffuse confluent multinodular masses with relatively homogeneous enhancement along the abdominal wall and the greater omentum and massive ascites (Fig. 1).

**Fig. 2:**
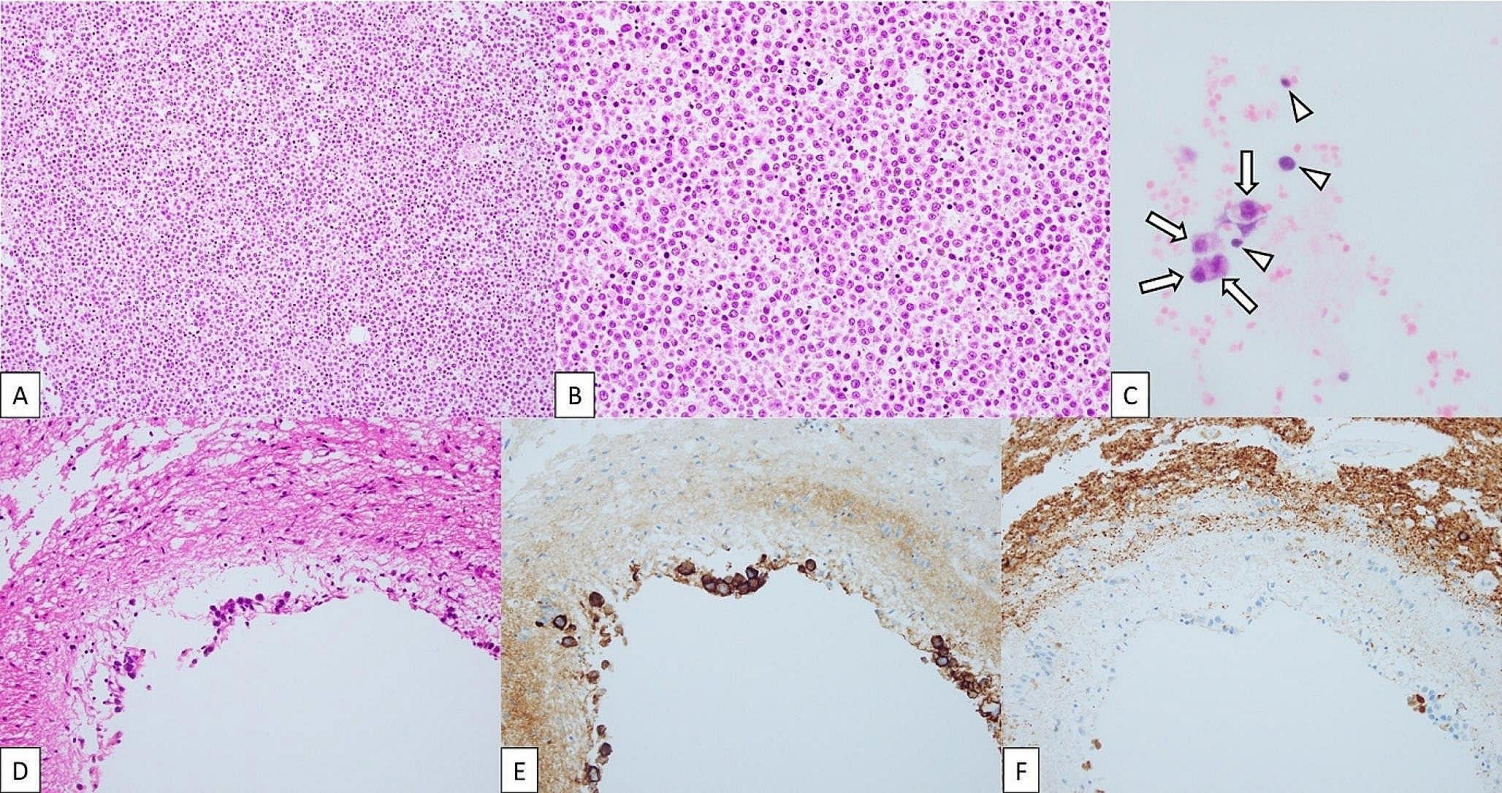
Autopsy pathology of the omental tumor (**A**, **B**), cell block specimen obtained from the VPS (**C**), and intracranial periventricular tumor (**D**–**F**). (**A**) HE stain (× 40) and (**B**) (× 100) showing diffuse proliferation of tumor cells with enlarged round to oval nuclei and clear to eosinophilic cytoplasm. (**C**) HE stain (× 400) reveals similar tumor cells (arrows) admixed with mesothelial cells (arrowheads) in the cell block specimen obtained from the VPS. (**D**) HE stain (× 100) shows similar tumor cells positive for D2-40 (**E**, × 200) and negative for calretinin (**F**, × 100). The tumor cells were histologically similar to those of the previously diagnosed pineal pure germinoma (not shown)

Serum tumor markers, including alpha-fetoprotein (AFP) and beta-human chorionic gonadotropin (hCG) were not elevated. Diagnostic paracentesis revealed class V cytology with bloody ascites; hence, drainage was deemed unsafe, with a high risk of anemia progression. The patient’s condition rapidly deteriorated, precluding chemotherapy, and he died one week after the CT examination. Autopsy revealed abdominal masses with histological features similar to those of a previous pineal pure germinoma (Fig. 2). Immunohistochemically, the tumor cells were negative for AE1/3, CK7, CK20, CDX-2, OCT3/4, CD30, AFP, and hCG and variably positive for Nanog, LIN28, PLAP, D2-40, and c-kit, consistent with germinoma, with a Ki-67 index of approximately 40%. The VPS contained tumor cells as well as mesothelial cells, suggesting the dissemination of pure germinoma, as well as retrograde flow into the VPS from the abdomen at the end of the disease.

## Discussion

Extraneural dissemination of primary central nervous system tumors is a rare occurrence after craniotomy or shunt placement, with documented incidence rates ranging from 0.1 to 3.7% of cases [7]. Extraneural dissemination of germinomas typically occur at a mean of 27 months (*n* = 25; 24 VPS + one ventriculoatrial-shunt case) after shunt placement [7]. Nearly all patients with extraneural dissemination had residual or recurrent intracranial tumors during shunt placement. In the present case, diffuse peritoneal dissemination with intraventricular recurrence was discovered 20 years post-VPS placement. Despite the absence of dissemination via the VPS at the initial onset and first recurrence, the rapid formation of diffuse peritoneal dissemination at the second recurrence may suggest biological transformation of the germinoma, including genomic and epigenomic alterations. Further studies are required to unravel the underlying mechanisms involved [2].

To our knowledge, there are no reports regarding abdominal imaging findings of peritoneal dissemination in pure germinoma cases. Referring to the limited reports on imaging findings in cases of mixed germ cell peritoneal dissemination, there is a tendency for multiple large masses with heterogeneous enhancement to form [8, 9]. In the present case, although hypovascular areas suggestive of central necrosis were observed in the largest mass, the masses showed homogeneous enhancement. Massive ascites accumulation was also a characteristic feature in the present case, which we believe was caused by the impaired absorption of cerebrospinal fluid flowing out of the VPS due to diffuse tumor dissemination. Given the shared morphology, immunophenotype, and molecular phenotype between germinoma and testicular seminoma (and ovarian dysgerminoma), the homogeneous contrast enhancement observed in the present case, as opposed to the heterogeneous enhancement observed in cases of peritoneal dissemination of mixed germ cell tumors, can be explained by the same mechanism underlying the tendency of seminomas to display homogeneous enhancement and non-seminomatous germ cell tumors to exhibit heterogeneous enhancement [1, 10]. In other words, the pathological differences between germinomas/ seminomas, which feature uniform tumor cell proliferation, and intracranial mixed germ cell tumors/ nonseminomatous germ cell tumors, which are more likely to be accompanied by areas of necrosis, hemorrhage, fibrosis, cystic changes, and calcifications, are reflected in radiological imaging findings.

## Conclusion

Although endoscopic third ventriculostomy rather than the VPS is now the recommended therapeutic method for hydrocephalus associated with germinomas, it is important to be aware of the possibility of peritoneal dissemination as a complication of long-term germinoma with VPS and associated imaging findings in patients with previously placed VPS.
